# Rootstock-regulated gene expression patterns associated with fire blight resistance in apple

**DOI:** 10.1186/1471-2164-13-9

**Published:** 2012-01-09

**Authors:** Philip J Jensen, Noemi Halbrendt, Gennaro Fazio, Izabela Makalowska, Naomi Altman, Craig Praul, Siela N Maximova, Henry K Ngugi, Robert M Crassweller, James W Travis, Timothy W McNellis

**Affiliations:** 1 Department of Plant Pathology, The Pennsylvania State University, University Park, PA 16802, USA; 2 The Pennsylvania State University Fruit Research and Extension Center, Biglerville, PA 17307, USA; 3 USDA/ARS, Plant Genetics Research Unit, Geneva, NY 14456, USA; 4 Institute of Molecular Biology and Biotechnology, Faculty of Biology, Adam Mickiewicz University, Poznan, Poland; 5 Department of Statistics, The Pennsylvania State University, University Park, PA 16802, USA; 6 Huck Institutes of the Life Sciences, The Pennsylvania State University, University Park, PA 16802, USA; 7 Department of Horticulture, The Pennsylvania State University, University Park, PA 16802, USA

## Abstract

**Background:**

Desirable apple varieties are clonally propagated by grafting vegetative scions onto rootstocks. Rootstocks influence many phenotypic traits of the scion, including resistance to pathogens such as *Erwinia amylovora*, which causes fire blight, the most serious bacterial disease of apple. The purpose of the present study was to quantify rootstock-mediated differences in scion fire blight susceptibility and to identify transcripts in the scion whose expression levels correlated with this response.

**Results:**

Rootstock influence on scion fire blight resistance was quantified by inoculating three-year old, orchard-grown apple trees, consisting of 'Gala' scions grafted to a range of rootstocks, with *E. amylovora*. Disease severity was measured by the extent of shoot necrosis over time. 'Gala' scions grafted to G.30 or MM.111 rootstocks showed the lowest rates of necrosis, while 'Gala' on M.27 and B.9 showed the highest rates of necrosis. 'Gala' scions on M.7, S.4 or M.9F56 had intermediate necrosis rates. Using an apple DNA microarray representing 55,230 unique transcripts, gene expression patterns were compared in healthy, un-inoculated, greenhouse-grown 'Gala' scions on the same series of rootstocks. We identified 690 transcripts whose steady-state expression levels correlated with the degree of fire blight susceptibility of the scion/rootstock combinations. Transcripts known to be differentially expressed during *E. amylovora *infection were disproportionately represented among these transcripts. A second-generation apple microarray representing 26,000 transcripts was developed and was used to test these correlations in an orchard-grown population of trees segregating for fire blight resistance. Of the 690 transcripts originally identified using the first-generation array, 39 had expression levels that correlated with fire blight resistance in the breeding population.

**Conclusions:**

Rootstocks had significant effects on the fire blight susceptibility of 'Gala' scions, and rootstock-regulated gene expression patterns could be correlated with differences in susceptibility. The results suggest a relationship between rootstock-regulated fire blight susceptibility and sorbitol dehydrogenase, phenylpropanoid metabolism, protein processing in the endoplasmic reticulum, and endocytosis, among others. This study illustrates the utility of our rootstock-regulated gene expression data sets for candidate trait-associated gene data mining.

## Background

Fire blight, the disease caused by the bacterial pathogen *Erwinia amylovora *(Burrill) [1], is a devastating, systemic disease that occurs in apples and other Rosaceous plants. Control is limited to pruning of infected branches and the use of antibiotics and copper compounds, both of which are only preventative, and are often strictly regulated. In addition, the emergence of streptomycin-resistant strains of *Erwinia amylovora *has raised questions about the continued use of this control agent [2]. Once established, infection leads to the development of necrotic regions on the leaves, shoots and petals. The infected regions of the plant eventually become brown or black and look as if swept by fire [3]. Severe fire blight outbreaks can result in the destruction of whole orchards. Current production methods have shifted towards high-density plantings on dwarfing or very-dwarfing rootstocks, resulting in greater yields per acre [4]. However many of the dwarfing rootstocks are highly susceptible to fire blight, resulting in greater disease problems.

The susceptibility of the different rootstocks and scion cultivars to fire blight varies substantially, and there are recommendations against certain combinations in regions particularly prone to fire blight [5]. It has been observed in the field and in the greenhouse that a given cultivar can have different levels of disease resistance depending on the rootstock to which it is grafted [6,7]. In the case of susceptible scion cultivars, it is recommended that they be grafted to resistant rootstocks to reduce susceptibility to fire blight. In the current study we demonstrate that rootstocks can have a significant effect on the resistance of the scion to fire blight.

A number of plant genes and pathways have been implicated as playing roles in the response to *E. amylovora *infection. Several *pathogenesis relate*d (*PR*) genes have been shown to be up-regulated in apple in response to *E. amylovora *infection [8,9]. Overexpression of *NPR1 *in apple results in increased *PR *gene expression and reduced susceptibility to *E. amylovora *and a number of other pathogens [10]. Norelli et al. [11] identified transcripts that are differentially expressed between control and *E. amylovora*-infected shoots using suppression subtractive cDNA hybridization. Recently, further studies identified additional transcripts that are differentially expressed during *E. amylovora *infection of apple leaves [12] and in apple flowers [13].

*E. amylovora *has been shown to specifically delay the expression of host genes in the phenylpropanoid pathway during infection [14,15]. This pathway leads to the production of anti-microbial compounds as well as lignin formation [16,17]. In addition, a general increase in free carbohydrate levels has also been associated with increased fire blight susceptibility [15].

Genetic analysis in apple is difficult due to its largely self-incompatible nature, high degree of heterozygosity, and large genome. However, the clonal propagation of apples provides an opportunity for genetic analysis of rootstock-regulated phenotypes, such as disease resistance. In a previous study, we used DNA microarrays to examine steady-state gene expression in the shoot tips of healthy, uninfected 'Gala' apple scions grafted to seven different rootstocks [18]. Each of the scion/rootstock combinations had a unique phenotype. In the present study, we undertook to identify constitutively expressed genes in 'Gala' apple whose expression levels were associated with a rootstock-induced decrease in fire blight susceptibility. Using fire blight resistance ratings from field-grown trees, we were able to mine the microarray data obtained during the earlier study [18] to identify genes and pathways that might be related to the tree fire blight susceptibility status. Previously, we used a similar approach to identify transcripts whose expression levels correlated with tree stature [18].

## Methods

### Plant Material

Trees for the fire blight tests were purchased from Adams County Nursery Inc. (Aspers, PA) and planted in five replica blocks at The Pennsylvania State University Fruit Research and Extension Center, Biglerville, PA. Trees consisting of 'Gala' scions grafted to a range of rootstocks were planted and conventionally managed with pesticides to control weeds, fungal diseases and insects in preparation for inoculation. For the greenhouse-grown tree first-generation microarrays, fresh bench grafts were grown as described previously [18]. For fire blight susceptibility tests, 'Crimson Gala' (Waliser cultivar) on the same seven rootstocks were planted in 2005 at the Fruit Research and Extension Center in Biglerville, PA. The rootstocks were, from the least to the most vigorous, Malling 27 EMLA (M.27), Budagovsky 9 (B.9), Malling 9 Fleuren 56 (M.9F56), Geneva 30 (G.30), Malling 7 EMLA (M.7), Supporter 4 (S.4), Malling Merton 111 EMLA (MM.111). Throughout the text, plants are described as scion/rootstock combinations. For example, a 'Gala' scion on an M.7 rootstock is designated as 'Gala'/M.7. The trees used for the second-generation microarray experiment were from a segregating population from an 'Ottawa 3' × 'Robusta 5' cross and were grown in an orchard in Geneva, NY [19]. This population had been previously characterized for resistance to fire blight [19].

### Fire Blight Susceptibility

For the fire blight tests conducted in Biglerville, PA, actively growing scion shoot tips of three-year old trees were wounded by using scissors to cut across the midribs of the youngest leaves, and a drop of phosphate buffer (10 mM, pH 7) containing 1 × 10^6 ^cfu/ml of *E. amylovora *(strain Ea581a or HKN06P1) was placed on the cut surface. The shoot tip was then covered for 24 hours with a plastic bag containing a wet piece of filter paper to maintain a humid environment and promote infection. Necrotic region measurements were taken over the course of the disease progression. Disease severity was calculated as the length of the blighted section of an inoculated shoot as a percentage of the total shoot length. Four replicate trials were conducted, each replicate consisting of 10 trees of each of the 7 scion/rootstock combinations. At least 5 shoots per tree were inoculated. The fire blight susceptibility of the 'Ottawa 3' × 'Robusta 5' cross progeny used for the second-generation microarray experiment, to two *E. amylovora *strains (Ea273, Ea2002a), was published previously [19]. The susceptibility data for a third *E. amylovora *strain (Ea4001a) is unpublished.

### RNA isolation and microarray analysis

The methods and results for our first-generation microarray, including RNA isolations from greenhouse-grown trees and microarray analysis, are described elsewhere [18]. The first generation array contained probes designed to detect 55,230 unique transcripts, representing up to 95% coverage of the apple genome. The sequences for all of the contigs used to develop the probes for the arrays can be found at the Gene Expression Omnibus (GEO) dataset website [20]. For the present study, we developed and used a second-generation apple DNA NimbleGen expression microarray that was designed based on our first-generation NimbleGen array [18] and used it to analyze RNA samples isolated from the progeny of the 'Ottawa 3' × 'Robusta 5' cross grown in Geneva, NY. The expression levels for each tree were analyzed on a single array only, with no biological replicates for any individual tree. The second-generation array was a 12-plex array containing 135,000 probes per plex, representing 26,017 transcripts, enabling us to query a relatively large number of samples. The probes for this array represent a subset of those included in the first-generation array. The second-generation array represents the transcripts with the best-performing probe sets from the first-generation array and includes the transcripts that showed differential expression between any two scion/rootstock combinations. Transcripts that had high variability among their probes were left off of the second-generation array. The five best-performing probes of the original six probes per transcript were used in the second-generation array to increase the number of different transcripts that could be queried by the second-generation array.

DNA microarray analysis on our second-generation microarrays was performed by the Penn State Genomics Core Facility at University Park, PA. Briefly, one microgram of total RNA from each sample was amplified using the Ambion (Life Technologies) Amino Allyl MessageAmp II aRNA Amplification Kit (AM1753) following the manufacturer's protocol for one cycle amplification. Fifteen micrograms of aRNA was dye coupled with either Cy3 or Cy5 (GE Health Care #RPN5661), as appropriate. Following quenching and cleanup of dye coupling reactions, 1.5 μg of a Cy3 labeled sample is combined with 1.5 μg of a Cy5 labeled sample and fragmented using RNA Fragmentation Reagents (Ambion AM8740) according the manufacturer's instructions. After fragmentation, samples are dried down completely in a speed-vac and then resuspended in tracking controls and hybridization solution according to the microarray manufacturer's instructions (Roche NimbleGen). Pairs of samples were hybridized overnight at 42°C with active mixing in a MAUI Hybridization System. Following hybridization, microarrays were washed and scanned according to the manufacturer's protocol (Roche NimbleGen). Images were burst, gridded, and pair files generated using NimbleScan software. The gene expression data from the hybridization experiments using the second-generation DNA microarray were normalized using R software and un-adjusted p-values were calculated by regression analysis using R software [21,22].

### Multiple regression analysis

Stepwise multiple regression analysis was used to identify genes whose expression levels were related to fire blight severity. The response variable was mean fire blight severity, calculated as the length of blighted section of an inoculated shoot as a percentage of the entire shoot and averaged across 5 replicates for each of 48 (Geneva trees). The explanatory variables were gene expression levels, expressed as arbitrary units of fluorescence intensity, for a set of 60 candidate genes selected based on potential for involvement in fire blight susceptibility. A full stepwise regression model was implemented so that each of the explanatory variables was evaluated in the regression with significance level for staying in the model set at *P *= 0.15. For both up-regulated and down-regulated candidate genes, separate stepwise regression analyses were computed for fire blight severity data for each of three *E. amylovora *strains. Stepwise multiple regression analysis were implemented using the REG procedure of SAS 9.2 (SAS Institute Inc., Cary, NC), and the resulting models were evaluated for goodness of fit based on standard regression analysis procedures [23].

## Results

### Rootstock-dependent differences in fire blight susceptibility of 'Gala' scions

**Figure 1:**
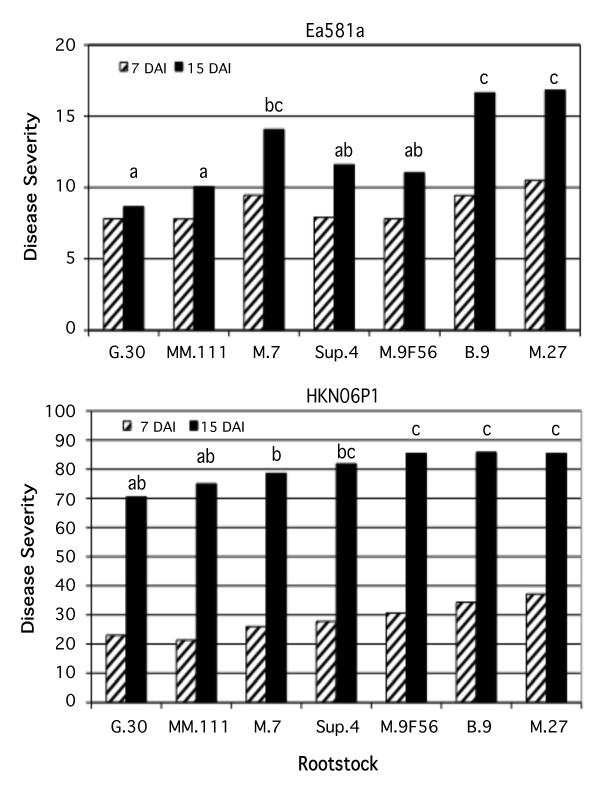
**Susceptibility of grafted 'Gala' scions on seven different rootstocks to two different strains of *E. amylovora***. Bars sharing the same letter are not significantly different (Fisher's LSD).

Significant differences in the relative size of the necrotic regions were observed within 15 days of inoculation of the 'Gala' shoot tips with two different strains of *E. amylovora *(Figure 1). For both strains, 'Gala'/G.30 and 'Gala'/M.111 were the least susceptible and 'Gala'/B.9 and 'Gala'/M.27 were the most susceptible. Interestingly, strain-dependent differences in fire blight susceptibility were observed for 'Gala'/M.7 and 'Gala'/M.9F56 trees. 'Gala'/M.7 susceptibility to *E. amylovora *strain Ea581a was similar to that of the most susceptible trees ('Gala'/B.9 and 'Gala'/M.27), while 'Gala'/M.9F56 susceptibility to strain Ea581a was similar to that of the most resistant trees ('Gala'/G.30 and 'Gala'/MM.111). The results were reversed with *E. amylovora *strain HKN06P1, with 'Gala'/M.7 susceptibility being similar to that of the most resistant trees and 'Gala'/M.9F56 susceptibility being similar to that of the most susceptible trees. *E. amylovora *strain Ea581a is a moderately virulent isolate, while HKN06P1 is a hypervirulent isolate [24].

### Clustering of trees according to phenylpropanoid pathway gene expression

After obtaining fire blight susceptibility ratings for all the 'Gala'/rootstock combinations used in the study, it became possible to ask whether expression levels of genes involved in specific biochemical pathways related to fire blight resistance might contribute to the rootstock-regulated fire blight resistance phenotype. Gene expression levels in scions of all the 'Gala'/rootstock combinations used in this study were previously profiled on a large scale using our first-generation DNA microarray [18], and these microarray data were mined in the present study for genes related to fire blight susceptibility. Because the phenylpropanoid biosynthesis pathway has been implicated in fire blight resistance [11,14], a search of the apple genome was conducted to find the predicted phenylpropanoid biosynthetic pathway genes. A total of 67 transcripts on our first-generation array were identified as belonging to the phenylpropanoid biosynthetic pathway, among other pathways (Additional File 1, Table S1).

**Figure 2:**
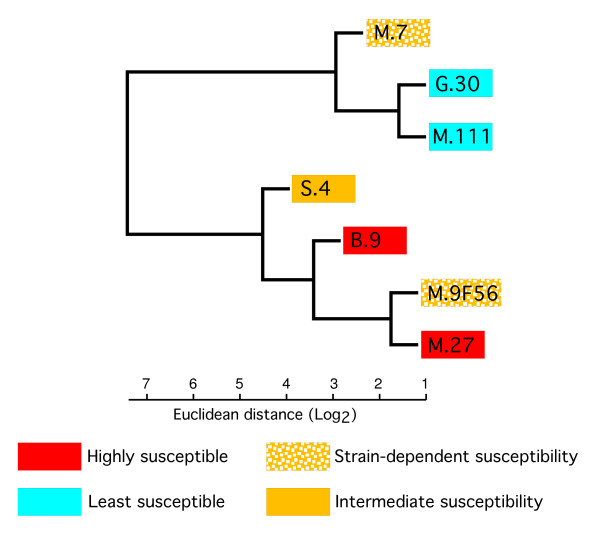
**Clustering analysis of transcripts involved in the phenylpropanoid biosynthetic pathway using complete linkage with Euclidean distance (Log_2_)**.

A complete linkage cluster analysis using the expression values for these genes was conducted to evaluate the potential contribution of the expression levels of genes in this pathway to resistance (Figure 2). There were two main clusters of trees based on the expression patterns of putative phenylpropanoid biosynthetic genes. The two least susceptible scion/rootstock combinations ('Gala'/G.30 and 'Gala'/M.111) were paired in one cluster, along with a branch containing one scion/rootstock combination displaying strain-dependent susceptibility ('Gala'/M.7). The second main cluster contained both of the highly susceptible scion/rootstock combinations ('Gala'/M.27 and 'Gala'/B.9) as well as a scion/rootstock combination with strain-dependent susceptibility ('Gala'/M.9F56) and the moderately susceptible scion/rootstock combination ('Gala'/S.4). Thus, the clustering of trees according to phenylpropanoid pathway gene expression closely followed the pattern of susceptibility to the highly virulent *E. amylovora *strain HKN06P1 (Figure 1).

### Clustering of trees according to sugar metabolic pathway gene expression

**Figure 3:**
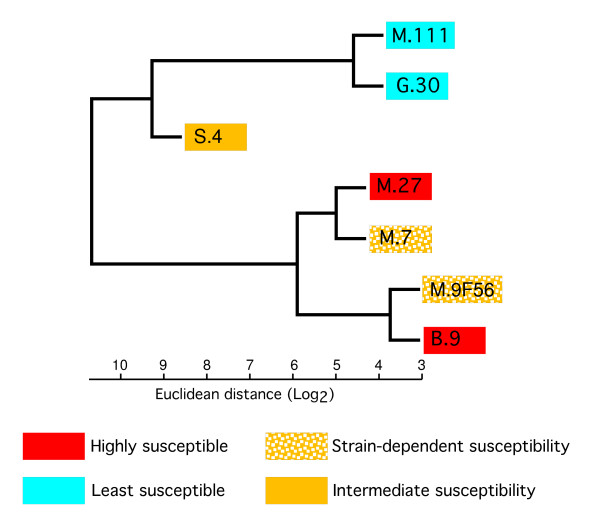
**Clustering analysis of transcripts involved in sugar metabolism using complete linkage with Euclidean distance (Log_2_)**.

The AraCyc metabolic pathways tool [25] was used to identify 93 Arabidopsis genes involved in sugar metabolism, and the potential homologs of these Arabidopsis genes in the apple genome were identified. A BLAST search of the set of the Arabidopsis genes involved in sugar metabolism to the apple genome resulted in the identification of 227 unique apple coding sequences. A total of 219 of these transcripts were represented on our first-generation array. A complete linkage cluster analysis using the expression values for the 219 identified sugar metabolism genes did not closely follow the pattern for the levels of fire-blight susceptibility for the various scion/rootstock combinations (compare Figures 1 &3). Instead, the clustering by sugar metabolism gene expression closely resembled clustering based on data for all the transcripts represented on the first-generation array [18].

### Identification and categorization of candidate rootstock-regulated, fire blight susceptibility-associated transcripts

**Figure 4:**
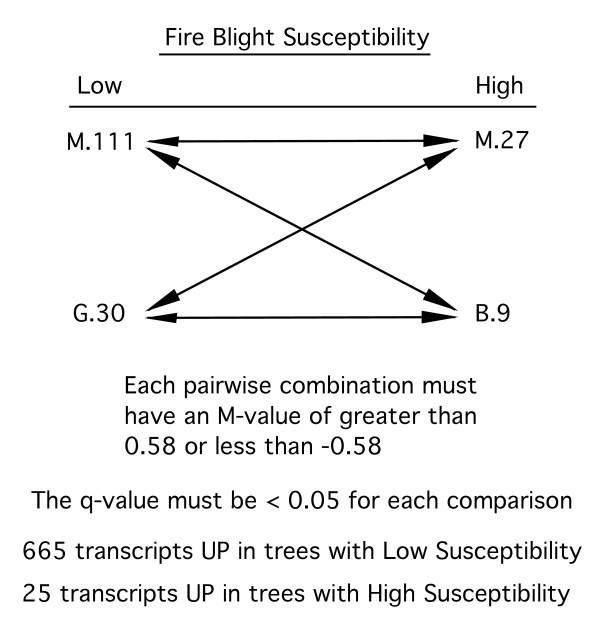
**Selection scheme used to identify candidate rootstock-regulated, fire blight susceptibility-associated transcripts based on expression data**.

Constitutive gene expression levels in scions of all the 'Gala'/rootstock combinations used in this study were profiled on a large scale previously using our first-generation apple DNA microarray [18]. Using the fire blight susceptibility ratings from the field trials, the microarray data were sorted to identify those transcripts whose expression levels correlated with the differences in fire blight susceptibility among the apple trees being studied. A diagram showing the comparisons used to sort the data is shown in Figure 4. Transcripts of interest were selected based on a fold-expression difference cutoff and a statistical strength cutoff. For a transcript to be selected, every possible pairwise comparison between the two least susceptible ('Gala'/G.30 and 'Gala'/M.111) and the two most susceptible scion/rootstock combinations ('Gala'/B.9 and 'Gala'/M/27) had to have at least a 1.5 fold difference in expression and a q-value of less than 0.05. This selection program resulted in a list of 665 transcripts with higher expression levels in the less susceptible trees (Additional File 1, Table S2), and 25 transcripts with higher expression in the more susceptible trees (Additional File 1, Table S3) for a total of 690 candidate rootstock-regulated, fire blight susceptibility-associated transcripts. The Malus genome BLAST hits and corresponding e-values are included in Additional File 1, Tables S2 and S3.

An analysis of the predicted functional categories of all of the candidate rootstock-regulated, fire blight susceptibility-associated transcripts was conducted based on the *Arabidopsis thaliana *BLASTX hits to the *Malus *x *domestica *sequence (BLASTX cutoff 1E-3). The distribution in the functional categories differed from that expected based on the whole transcriptome. These Gene Ontology (GO) enrichment patterns are shown in Table 1. Transcripts of genes predicted to be involved in responses to stress and biotic and abiotic stimuli were disproportionately represented relative to the known apple transcriptome as a whole.

**Table 1:** GO Enrichment Analysis

Keyword Category	Functional Category	Expected	Observed
Biological Process	cell organization and biogenesis	29	29
Biological Process	developmental processes	22	21
Biological Process	DNA or RNA metabolism	8	2
Biological Process	electron transport or energy pathways	13	21
Biological Process	**other biological processes**	20	37*
Biological Process	other cellular processes	138	164
Biological Process	other metabolic processes	146	162
Biological Process	protein metabolism	60	60
Biological Process	**response to abiotic or biotic stimulus**	23	53*
Biological Process	**response to stress**	19	46*
Biological Process	signal transduction	15	7
Biological Process	**transcription**	23	4*
Biological Process	transport	31	31
Biological Process	**unknown biological processes**	124	33*
		
		670	670

*Chi Square < 0.001

A functional annotation of the transcripts in Additional File 1, Table S2 was conducted using the Kyoto Encyclopedia of Genes and Genomes (KEGG) [26] to look for pathways that might be overrepresented among the candidate rootstock-regulated, fire blight susceptibility-associated transcripts. The sequence of every transcript on the array was subjected to KEGG analysis to generate an overall picture of the relative abundance of genes in the KEGG pathways. Similarly, all of the transcripts in Additional File 1, Tables S2 and S3 were analyzed for the KEGG pathways. A chi-square analysis revealed that, for the candidate rootstock-regulated, fire blight susceptibility-associated transcripts, several pathways contained more genes than predicted relative to a KEGG analysis of all of the transcripts represented on the array (Table 2). Those pathways that had the highest confidence level for being over-represented include: fatty acid metabolism (ko00071), valine, leucine and isoleucine degradation (ko00280), photosynthesis-antenna proteins (ko00196), flavonoid biosynthesis (ko00941), protein processing in the endoplasmic reticulum (ko04141), endocytosis (ko4144) and peroxisome (ko4146).

**Table 2:** KEGG functional analysis of candidate rootstock-regulated, fire blight susceptibility-associated transcripts

		Whole array	Suppl Table 2		Table 3 transcripts		Table 5 transcripts		p-value >		
**Pathway**	**KEGG no**.	**Total**	**Exp**	**Obs**	**Exp**	**Obs**	**Exp**	**Obs**	**Suppl Table 2**	**Table 3**	**Table 5**

Fatty acid metabolism	71	10	0.1	4	n/a	n/a	n/a	n/a	0.001	n/a	n/a

Protein processing in endoplasmic reticulum	4141	60	0.8	8	0.3	3	0.3	3	0.001	0.001	0.001

Carbon fixation pathways in prokaryotes	720	10	0.1	3	n/a	n/a	n/a	n/a	0.001	n/a	n/a

Peroxisome	4146	30	0.4	5	0.2	1	n/a	n/a	0.001	0.05	n/a

Photosynthesis-antenna proteins	196	12	1.5	7	0.7	1	0.7	2	0.001	NS	0.001

Endocytosis	4144	30	0.4	3	0.2	2	0.2	1	0.001	0.001	0.05

Valine, leucine and isoleucine degradation	280	20	0.3	2	n/a	n/a	n/a	n/a	0.001	n/a	n/a

Carbon fixation in photosynthetic organisms	710	22	2.8	8	1.3	2	n/a	n/a	0.01	NS	n/a

Methane metabolism	680	19	2.4	7	1.1	2	n/a	n/a	0.01	NS	n/a

Spliceosome	3040	95	12.2	3	5.5	1	5.2	1	0.01	NS	NS

Arginine and proline metabolism	330	30	0.4	2	n/a	n/a	n/a	n/a	0.01	n/a	n/a

Flavonoid biosynthesis	941	13	1.7	5			0.7	2	0.01		NS

Antigen processing and presentation	4612	6	0.8	3	0.3	2	0.3	2	0.05	0.01	0.01

Purine metabolism	230	75	9.6	2	n/a	n/a	n/a	n/a	0.05	n/a	n/a

Pentose and glucuronate interconversions	40	10	0.1	1	n/a	n/a	n/a	n/a	0.05	n/a	n/a

Indole alkaloid biosynthesis	901	1	0.1	1	n/a	n/a	n/a	n/a	0.05	n/a	n/a

Neuroactive ligand-receptor interaction	4080	1	0.1	1	n/a	n/a	n/a	n/a	0.05	n/a	n/a

Renin-angiotensin system	4614	1	0.1	1	n/a	n/a	n/a	n/a	0.05	n/a	n/a

Benzoate degradation	362	4	0.5	2	n/a	n/a	0.3	1	0.05	n/a	NS

Fructose and mannose metabolism	51	16	2.1	5	0.9	1	n/a	n/a	0.05	NS	n/a

Ubiquitin mediated proteolysis	4120	47	6.0	1	n/a	n/a	3.2	1	0.05	n/a	NS

Cell cycle-yeast	4111	46	5.9	1	n/a	n/a	3.1	1	0.05	n/a	NS

Phagosome	4145	26	3.3	7	n/a	n/a	1.4	3	0.05	n/a	NS

NOD-like receptor signaling pathway	4621	3	0.4	1	0.2	1	0.2	1	NS	0.05	NS

NS = not significant, n/a = no transcripts identified matching this pathway.

### Genes differentially expressed during fire blight infection are disproportionately represented among the candidate rootstock-regulated, fire blight susceptibility-associated transcripts

Transcripts that increase or decrease in abundance during fire blight infection have been identified in apple [11-13]. Of the 690 candidate, rootstock-regulated, fire blight susceptibility-associated transcript sequences identified in the present study (Additional File 1, Tables S2 & S3), 79 (Table 3) had been determined to be differentially expressed during fire blight infection by Norelli et al., [11]. Based on BLAST searches of both sets of transcripts to the recently published apple genome [27], a number of the transcript sequences were found to represent different locations on the same predicted gene, resulting in a final set of 54 unique genes (Table 3). Based on the sizes of the candidate gene lists and the number of genes on the array and in the genome, approximately four genes are expected to be in common between the two data sets due to chance.

**Table 3:** Transcripts in common with those found to have differential expression in apple upon *E. amylovora *infection by Norelli et al., [11]

Seq_ID	GenBank_Accn	Malus Contig BLAST Hit	Description	evalue	SSH Response^§^
APPLE0F000017044/	EG974017	MDC011793.214	plastocyanin-like domain-containing protein	9E-14	early up-regulated
APPLE0FR00078469					

APPLE0F000011437/			fibrillarin 1 (FBR1) (FIB1) (SKIP7) identical to fibrillarin 1	1E-59	
APPLE0F000051475/	EG974020	MDC019359.48	GI:9965653 from [A. thaliana]; C-terminus identical to SKP1	3E-53	early up-regulated
APPLE0F000020924			interacting partner 7 GI:10716959 from [A. thaliana]	3E-28	

APPLE0F000022573	EH009495	MDC009164.88	putative cytochrome c oxidase subunit 5c [Helianthus annuus]	5E-14	up-regulated 1 hpi

*APPLE0F000017734/*	EH009489	MDC002049.218	leucine-rich repeat transmembrane protein kinase, putative	4E-11	up-regulated 1 hpi
*APPLE0F000060312*				9E-06	

APPLE0F000019504	EH009551	MDC011946.321	L-ascorbate peroxidase 1, cytosolic (APX1	7E-12	up-regulated 1 hpi

APPLE0FR00043470	EH009570	MDC010527.333	Encodes a novel component essential for NDH-mediated non-photochemical reduction of the plastoquinone pool in chlororespiratory electron transport.		up-regulated 1 hpi

APPLE0F000017575/	EH009572	MDC015841.261	oxygen-evolving enhancer protein 3, chloroplast, putative	5E-13	up-regulated 1 hpi
APPLE0F000020381			(PSBQ2	7E-11	

**APPLE0F000015792**	EH009577	MDC006495.372	catalase 3 (SEN2)	3E-12	up-regulated 1 hpi
**APPLE0F000015821**				4E-16	

APPLE0F000006776	EH009561/	MDC013772.152	putative strong similarity to plastidic fructose-bisphosphate)	3E-25	up-regulated 1 hpi
	EH009491		aldolase (EC 4.1.2.13) from Nicotiana paniculata (NPALDP1		

APPLE00R00054041	EH009563	MDC008063.227	similar to putative 60S acidic ribosomal protein P0 GB:P50346	4E-32	up-regulated 1 hpi

APPLE0F000022368	EH090785	MDC016063.220	Glycine-rich RNA-binding protein 7 {A. thaliana}	2E-11	up-regulated 12 hpi

APPLE00R00018459	EH090787	MDC002341.180	40S ribosomal protein S27 (ARS27A	2E-17	up-regulated 12 hpi

APPLE0F000016047/	EH090788	MDC013560.291	chlorophyll A-B binding protein 2, chloroplast/LHCII type I	5E-27	up-regulated 12 hpi
APPLE0F000021251			CAB-2/CAB-140 (CAB2B)		

**APPLE00R00018530**	EH090762	MDC009496.462	putative mRNA binding protein precursor (GI:26453355)	1E-22	up-regulated 2 hpi

**APPLE0F000021275**	EH090763	MDC011225.39	dehydrin ERD10 (Low-temperature-induced protein LTI45) [A. thaliana] SWISS-PROT:P42759		up-regulated 2 hpi

APPLE0F000020273	EH090764	MDC007862.188	heat shock protein 81-4 (HSP81-4) contains Pfam profiles PF02518, PF00183: Hsp90 pro	1E-22	up-regulated 2 hpi

APPLE0F000017683/	EG974803	MDC009596.270	sugar transporter, putative similar to ERD6 protein {A. thaliana}	8E-29	up-regulated 48 and 72 hpi
APPLE0F000019355				1E-33	72 hpi

APPLE0F000015156	EG974779	MDC021689.444	contains Pfam profile PF00407: Pathogenesis-related protein Bet v I family	1E-11	up-reg. 48 and 72 hpi

**APPLE0F000018467/**					
**APPLE0F000019900/**					
**APPLE0F000060942/**	EG974812	MDC007381.261	cytosolic (GAPC)/NAD-dependent glyceraldehyde-3-phosphate	1E-20	up-reg. 48 and 72 hpi
***APPLE0FR00073702/***			dehydrogenase		
***APPLE0FR00045573/***					

APPLE0F000015746	EG974761	MDC017137.174	ribulose bisphosphate carboxylase small chain 3B/RuBisCO small subunit 3B (RBCS-3B) (ATS3B	8E-20	up-reg. 48 and 72 hpi

APPLE00R00062179	EH034496/	MDC006746.407	conserved hypothetical protein [Corynebacterium efficiens YS-	4E-07	early up-reg., down-,
	EG974019		314] mitochondrial 26S ribosomal RNA protein		reg. 2 hpi

*APPLE00R00062069/*					
*APPLE00R00062142/*					early up-reg., up-reg.
*APPLE0FR00063223/*	EH009493/	MDC021568.167	3'utr of MDP0000053760 Unknown A. thaliana. protein	3E-12	1 hpi, down-reg. 2
*APPLE0FR00072001/*	EG974018				hpi,
*APPLE0FR00074986*					

APPLE0F000019494/	EH034531/	MDC008933.302	jasmonate-zim-domain protein 1		up-reg. 1 hpi, down-
APPLE0FR00070531	EH009567				reg. 2 hpi

APPLE0F000015761/	EH034470/			1E-20	
APPLE0F000016441/	EH034448/	MDC012615.331	chlorophyll A-B binding protein 2, chloroplast/LHCII type I	3E-70	up-reg. 1 hpi, down-
APPLE0F000017178/	EH009539/		CAB-2/CAB-140 (CAB2B)	1E-20	reg. 2 hpi,
APPLE0F000016322	EH009499			1E-70	

	EH034678/				
	EH034677/				
	EH034635/				up-reg. 1 hpi, down-
APPLE00R00015736/	EH034631/	MDC017026.232	ribulose bisphosphate carboxylase small chain 3B/RuBisCO	4E-26	reg. 1 hpi, down-reg.
APPLE0F000025537	EH034620/		small subunit 3B (RBCS-3B) (ATS3B)		12 hpi, down-reg. 24
	EH034600/				hpi, down-reg. 48
	EH034581/				hpi,
	EH034578/				
	EH009517/				
	EH009492				

APPLE00R00061494	EH034644	MDC008988.418	ribosomal protein L2 family protein similar to ribosomal protein L2 [Gossypium arboreum] GI:17644114; contains Pfam profile PF03947: Ribosomal Proteins L2, C-terminal domain	4E-07	down-regulated 1 hpi

APPLE00R00016015	EH034651	MDC013883.400	glycosyl hydrolase family 1 protein contains Pfam PF00232: domain; TIGRFAM TIGR01233: 6-phospho-beta-galactosidase; similar to amygdalin hydrolase isoform AH I precursor	1E-22	down-regulated 1 hpi

APPLE0F000019334	EH034681	MDC023674.27	expressed protein	4E-25	down-regulated 1 hpi

APPLE0F000015900	EH034690	MDC013694.297	68414.m02538 plastocyanin similar to plastocyanin GI:1865683 from [A. thaliana]	9E-11	down-regulated 1 hpi

	EH034668/				
APPLE0FR00069028	EH034488/	MDC019115.109	piggyBac transposable element derived 5 [Homo sapiens]	1E+00	down-reg. 1 hpi,
	EH034454				down-reg. 2 hpi

**APPLE0F000059582**	EH034691/	MDC011711.503	elongation factor 1-alpha/EF-1-alpha	1E-81	down-reg. 1 hpi,
	EH034556				down-reg. 24 hpi

APPLE0F000021983	EH034459/	MDC004849.508	sodium/calcium exchanger family protein/calcium-binding EF	2E-18	down-regulated 2 hpi
	EH034663		hand family protein contains Pfam profiles: PF01699		

APPLE0F000017271	EH034459/	MDC004849.509	oxygen-evolving enhancer protein, chloroplast, putative/33 kDa	3E-48	down-regulated 2 hpi
	EH034663		subunit of oxygen evolving system of photosystem II,		

APPLE0F000022030	EH034485	MDC015890.82	ADP-ribosylation factor, putative similar to DcARF1	3E-26	down-regulated 2 hpi

APPLE0F000018523	EH034540	MDC015915.252	heat shock protein 70, putative/HSP70, putative strong similarity to heat shock protein GI:425194 [Spinacia oleracea]	8E-49	down-regulated 2 hpi

APPLE0F000020112	EH034525	MDC000302.731	ribulose bisphosphate carboxylase small chain 3B/RuBisCO small subunit 3B (RBCS-3B) (ATS3B)	7E-06	down-regulated 2 hpi

APPLE0F000050108/					
APPLE0F000061371/	EH034439	MDC021556.182	polyubiquitin (UBQ4) identical to GI:17677	1E-112	down-regulated 2 hpi
APPLE0F000022561					

APPLE0F000060823	EH034517	MDC012000.76	heat shock cognate 70 kDa protein 1 (HSC70-1) (HSP70-1)		down-regulated 2 hpi

APPLE0F000017293	EH034563/	MDC021812.77	peroxidase 42 (PER42) (P42) (PRXR1	2E-57	down-reg. 2 hpi,
	EH034487				down-reg. 24 hpi

APPLE0F000017691	EH034538	MDC021024.27	phosphatidylinositol-4-phosphate 5-kinase family protein	4E-51	down-reg. 24 hpi

APPLE0FR00045757	EH034536	MDC016554.129	possible 5' utr for Thiamin diphosphate-binding fold (THDP-binding) superfamily protein		down-reg. 24 hpi

APPLE00R00002434/	EH034628	MDC008650.425	germin-like protein (GER3)	1E-62	down-reg. 48 hpi
APPLE0F000049504				9E-08	

APPLE0F000018527	EH034604	MDC005688.181	oxygen-evolving enhancer protein, chloroplast, putative/33 kDa subunit of oxygen evolving system of photosystem II		down-reg. 48 hpi

APPLE00R00058028	EH034627	MDC010241.225	putative/L-iditol 2-dehydrogenase, putative similar to NAD-dependent sorbitol dehydrogenase from Malus x domestica	9E-26	down-reg. 48 hpi

APPLE0FR00038628	EH009528	MDC000300.335	No Hits Found		up-regulated 1 hpi

APPLE0F000015847/	EH034662	MDC022487.75	chlorophyll A-B binding protein/LHCII type I (LHB1B1)	5E-31	
APPLE0F000016638				4E-40	

APPLE0FR00073381	EH034693	MDC000997.260	No Hits Found		

APPLE0F000024130	EH034466	MDC004582.94	protochlorophyllide reductase A, chloroplast/PCR A/NADPH-protochlorophyllide oxidoreductase A	5E-9	

APPLE0F000059277	EG974791	MDC004462.498	60S ribosomal protein L19 (RPL19C) similar to L19 from several species	4E-41	

APPLE0FR00073380	EH034693	MDC000997.260	No Hits Found		

APPLE0FR00039729	EH009538	MDC005169.268	No Hits Found		

APPLE0FR00036633	EH009530	MDC001354.357	hypothetical protein [Burkholderia fungorum]	.02	

Up in sensitive					

APPLE0F000061746	EG974808/	MDC022200.129	catechol oxidase activity molecular function Catalysis of the reaction: 2 catechol + O2 metal ion binding molecular function		up-reg. 48 and 72 hpi
	EG974767		Interacting selectively with any metal ion. predicted with glimmer		

APPLE0F000001556	EH034619	MDC011650.665	expressed protein protein induced upon wounding-A. thaliana	4E-79	down-regulated 48 hpi

§ SSH response corresponds to the change in expression after infection.

Bold text corresponds to genes that are phosphorylated upon infection.

Italicized text corresponds to genes that were also identified as differentially expressed upon infection by Baldo et al., [12].

Underlined text corresponds to genes that were also identified as differentially expressed upon infection by Sarowar et al., [13].

Similarly, a total of 20 of our candidates corresponded to 10 genes identified by Baldo et al., [12] (Table 4), out of a total of 190 genes identified in that study. Half of these 20 candidates were also among those identified by Norelli et al [11]. We also compared our candidate list to a list of ~3,500 genes identified as being differentially expressed during flower infection by Sarowar et al., [13] and found that 117 of our candidates shared an Arabidopsis BLAST hit (Additional File 1, Table S4). For Baldo et al., and Sarowar et al., the number of genes expected to be in common between the two data sets due to chance are 2 and 60 respectively.

**Table 4:** Transcripts in common with those found to have differential expression in apple upon *E. amylovora *infection by Baldo et al., [12]

SEQ_ID	GenBank_Accn	Malus Contig BLAST Hit	Description	evalue	cDNA-AFLP response^§^
*APPLE0F000017734/*	EX982051	MDC002049.218	leucine-rich repeat transmembrane protein kinase, putative"	4E-11	up-regulated 2
*APPLE0F000060312*				9E-06	hpi in M.26

*APPLE00R00062069/*					
*APPLE00R00062142/*					
*APPLE0FR00063223/*	EX982063	MDC021568.167	235aa long hypothetical protein [Pyrococcus horikoshii]	3E-12	up-regulated 2
*APPLE0FR00072001/*			hypothetical protein [Deinococcus radiodurans]	5E-11	hpi in M.26
*APPLE0FR00074986*					

*APPLE00R00062069/*					
*APPLE00R00062142/*					
*APPLE0FR00063223/*	EX982064	MDC021568.167	235aa long hypothetical protein [Pyrococcus horikoshii]	3E-12	down-regulated
*APPLE0FR00072001/*			hypothetical protein [Deinococcus radiodurans]	5E-11	2 hpi in M.26
*APPLE0FR00074986*					

*APPLE00R00062069/*					
*APPLE00R00062142/*					
*APPLE0FR00063223/*	EX982070	MDC021568.167	235aa long hypothetical protein [Pyrococcus horikoshii]	3E-12	up-regulated 2
*APPLE0FR00063223/*			hypothetical protein [Deinococcus radiodurans]	5E-11	& 48 hpi
*APPLE0FR00072001/*					in M.26
*APPLE0FR00074986*					

APPLE0F000062056	EX982066	MDC001431.265	hypothetical protein [Oenothera elata subsp. hookeri]	9E-18	up-regulated 2 hpi in G-41

*APPLE0FR00045573/*	EY437146	MDC005648.389			up-regulated 48
*APPLE0FR00073702*					hpi in G-41

APPLE0F000020631/	EX982080	MDC005535.336	invertase/pectin methylesterase inhibitor family protein similar to pectinesterase from Arabidosis thaliana	2E-19	up-regulated 48 hpi in G-41

APPLE0F000016328/			plasma membrane intrinsic protein, putative very strong	7E-57	
APPLE0FR00076854/	EX982085	MDC003306.225	similarity to plasma membrane intrinsic protein (SIMIP)		up-regulated 48
APPLE0FR00076855			[Arabidopsis thaliana] GI:2306917"		hpi in G-41

APPLE00R00015876/				3E-19	
APPLE0F000019554/				4E-11	
APPLE0F000021355/	EX982096	MDC012593.380	chlorophyll A-B binding protein (LHCB2:4) nearly	7E-16	up-regulated 48
APPLE0F000024114/			identical to Lhcb2 protein [Arabidopsis thaliana]	5E-06	hpi in G-41
APPLE0FR00036952/			GI:4741950		
APPLE0FR00037537					

APPLE0F000062056	EX982108	MDC001431.265	hypothetical protein [Oenothera elata subsp. hookeri] 3' UTR of MDP499035	9E-18	down-regulated 48 hpi in G-41

§ cDNA-AFLP response corresponds to the change in expression after infection

Bold text corresponds to genes that are phosphorylated upon infection

Italicized text corresponds to genes that were also identified as differentially expressed upon infection by Norelli et al., [11]

Underlined text corresponds to genes that were also identified as differentially expressed upon infection by Sarowar et al., [13].

Interestingly, of the 54 transcripts identified in common with Norelli et al., over half were down-regulated post infection [11]. However, we found that these same genes had higher expression levels in the less susceptible trees. A few of these were initially up-regulated in the Norelli study at early time points, but were down-regulated at later time points. In Sarowar et al., again over half of the transcripts in common were identified as being down-regulated upon infection; they were expressed at higher levels in the least susceptible trees in the present study. Only 2 of 10 of the genes in common with Baldo et al. were down-regulated upon infection. A KEGG analysis of the genes in Table 3 is included in Table 2. Those pathways that had the highest confidence level for being overrepresented relative to the total transcriptome include protein processing in the endoplasmic reticulum (ko04141) and endocytosis (ko4144).

### Analysis of expression patterns of candidate fire blight susceptibility-associated genes in an apple rootstock breeding population

To further analyze which candidate rootstock-regulated, fire blight susceptibility-associated transcripts might prove to be the best indicators of resistance, we examined the expression levels of a subset of the candidate transcripts in a test population of 48 individual, non-grafted apple lines, grown in Geneva, NY, that were offspring from a single 'Ottawa 3' × 'Robusta 5' cross segregating for fire blight resistance. Expression was measured using a second-generation, 135,000 feature microarray (representing ~26,000 transcripts) developed from the original, larger microarray used for rootstock-regulated gene expression profiling [18]. The second-generation microarray was designed before the 690 fire blight-associated candidate transcripts had been identified; of the 690 candidate transcripts, 429 were represented on the second-generation microarray.

Three different strains of *E. amylovora *(Ea2002a, Ea273a, and Ea4001a) were used to determine the susceptibility of the segregating population. Regression analysis of the data identified a set of 39 transcripts out of the 429 candidates that had some association with fire blight resistance in the breeding population (Table 5). The transcripts included in this set had un-adjusted p-values of less than 0.05.

**Table 5:** Candidate transcripts whose expression levels correlated with fire blight resistance in a population of trees segregating for fire blight susceptibility (regression analysis).

	*E. amylovora *strain					
**Seq_ID**	**E2002a**	**Ea273**	**4001a**	**hit**	**Description**	**evalue**

APPLE0F000021750		X	X	At3g54020	inositol phosphorylceramide synthase 2 -	3E-19

APPLE0F000017942		X	X	At5g17420	cellulose synthase, catalytic subunit (IRX3) identical to gi:5230423	5E-08

APPLE00R00017800	X		X	At5g02570	histone H2B, putative similar to histone H2B-2 Lycopersicon esculentum	2E-24

APPLE0F000016970	X			At3g01090	Snf1-related protein kinase (KIN10) (SKIN10)	2E-14

APPLE0F000020273*	X			At5g56000	heat shock protein 81-4 (HSP81-4)	

APPLE0FR00077763	X			No Hits Found		1E-34

APPLE0F000025563	X			At3g47520	malate dehydrogenase [NAD], chloroplast (MDH) identical to chloroplast NAD-malate dehydrogenase [A. thaliana] GI:3256066	9E-30

APPLE0FR00039135	X			No Hits Found		

APPLE0F000018055		X		At3g22550	similar to senescence-associated protein SAG102	4E-41

APPLE0FR00077391		X		No Hits Found		1E+00

APPLE0F000059277		X		At4g02230	60S ribosomal protein L19	

APPLE0FR00069907		X		No Hits Found		2E-18

APPLE0F000018558		X		At3g47470	chlorophyll A-B binding protein 4, LHCI type III CAB-4 (CAB4)	1E-22

APPLE0FR00078469		X		No Hits Found		

APPLE0FR00072723		X		gb|AAD45359|AF161252_1	cycloidea-like protein [Linaria vulgaris]	2E-06

APPLE0FR00048629		X		emb|CAG59326||	unnamed protein product [Candida glabrata CBS138] ref|XP_4463991| unnamed protein product [Candida glabrata]	2.E-02

APPLE0FR00038610		X		No Hits Found		

APPLE00R00062246		X		dbj|BAB1270|	P0671B1122 [Oryza sativa (japonica cultivar-group)]	2.E-06

APPLE0F000021761		X		At1g72370	40S ribosomal protein SA (RPSaA) identical to laminin receptor-like protein GB:U01955 [A thaliana]	9.E-20

APPLE0FR00036516			X	gb|EAA23003|	Ribosomal protein L31e, putative [Plasmodium yoelii yoelii]	2E-06

APPLE0FR00076821			X	No Hits Found		

APPLE0FR00076762			X	No Hits Found		

APPLE0F000016441*			X	At1g29930	chlorophyll A-B binding protein 2, LHCII type I CAB-2/CAB-140	3.E-70

APPLE0F000019498			X	At2g42210	mitochondrial import inner membrane translocase subunit Tim17/Tim22/Tim23 family protein	8E-07

APPLE0F000018523*			X	At3g12580	heat shock protein 70, putative/HSP70, putative	8E-49

APPLE00R00018643			X	At3g27690	chlorophyll A-B binding protein (LHCB2:4)	4E-22

APPLE0F000020073			X	At2g34690	expressed protein	3E-14

APPLE0F000021409			X	At3g05890	hydrophobic protein (RCI2B)/low temperature and salt responsive protein (LTI6B)	5E-10

APPLE0FR00037149			X	No Hits Found		

APPLE0FR00080491			X	gb|AAK07949|AF318573_29	unknown [Bovine herpesvirus 4]	1E+00

APPLE0F000025192			X	At3g56900	aladin-related/adracalin-related weak similarity to SP|Q9NRG9	3E-16

APPLE0F000060354			X	At5g02120	68418m00133 thylakoid membrane one helix protein (OHP)	1E+00

APPLE0F000001282			X	At4g38920	vacuolar ATP synthase 16 kDa proteolipid subunit 3/V-ATPase	1.E-34

APPLE0F000058071			X	At1g75780	tubulin beta-1 chain (TUB1) nearly identical to SP|P12411	2E-02

APPLE0F000020583			X	At2g34250	protein transport protein sec61, putative similar to PfSec61 [Plasmodium falciparum] GI:3057044	6E-34

APPLE0F000019736			X	At5g39740	60S ribosomal protein L5 (RPL5B) ribosomal protein L5, rice	2E-02

APPLE0FR00045816			X	No Hits Found		

APPLE0FR00036516			X	gb|EAA23003|	Ribosomal protein L31e, putative [Plasmodium yoelii yoelii]	1E-16

APPLE0F000018069			X	At5g09810	actin 7 (ACT7)/actin 2 identical to SP|P53492 Actin 7 (Actin-2) {Arabidopsis thaliana}	4E-72

APPLE0F000026657^#^		X		At1g04540	C2 domain-containing protein low similarity to cold-regulated gene SRC2 [Glycine max]	3E-11

APPLE0FR00032503^#^		X		ref|XP_032996|	similar to KIAA0819 protein [Homo sapiens]	3E-01

APPLE0F000061746^#^	X			sp|P43309|PPO_MALDO	Polyphenol oxidase, chloroplast precursor (PPO) (Catechol oxidase)	0E+00

X = an un-adjusted p value of less than 0.05 for the analysis

* Transcripts also found in Table 3

^# ^Transcripts less abundant in plants with low susceptibility to fire blight

Underlined text corresponds to genes that were also identified as differentially expressed upon infection by Sarowar et al., [13].

For the transcripts identified as having higher levels of expression in less susceptible trees, 3 had an un-adjusted p-value below 0.05 for two strains, and 36 had an un-adjusted p-value below 0.05 for one strain. For those transcripts that had higher expression levels in the more susceptible scion/rootstock combinations, only 3 transcripts had un-adjusted p-values below 0.05. The transcript list in Table 5 includes 3 transcripts previously shown to be differentially expressed upon *E. amylovora *infection [11] (Table 3). The functional annotation of these genes is included in Table 2. Those pathways, as determined by the KEGG analysis, that had the highest confidence level for being disproportionately represented include: photosynthesis-antenna proteins (ko00196), protein processing in the endoplasmic reticulum (ko04141), and endocytosis (ko4144).

### Gene expression patterns correlating with fire blight susceptibility

A total of 13 genes with higher transcript levels in more resistant trees were identified as being significantly related to the level of fire blight caused by at least one of the three *E. amylovora *strains, with the resulting regression models accounting for between 40 and 60% of the variation in disease severity (Additional File 2, Table S5). Of these, increased transcription of one gene (APPLE0F000020273) was negatively related to levels of fire blight caused by all three strains. Increased levels of transcripts of APPLE0F000027501, APPLE00R00018643 and APPLE0F000019968 were related to lower levels of fire blight caused by at least two of the *E. amylovora *strains, while increased transcript levels of APPLE0F000019334, APPLE0F000018558, APPLE0F000020583 and APPLE0F000023953 were noted in tissues with higher levels of disease for at least two of the strains (Additional File 2, Table S5).

The models relating the expression of genes with higher levels of expression in more susceptible trees to levels of fire blight were much weaker, only accounting for between 29 and 32% of disease severity (0.031 ≤ *P *≤ 0.009; Additional File 2, Table S6). In all, lower expression levels of eight of the genes were related to levels of fire blight for at least one of the *E. amylovora *strains, with APPLE0FR00081295, APPLE0FR00067567 and APPLE0F000016771 being related with higher levels of fire blight caused by Ea 273 (Additional File 2, Table S6). Only the lower transcript levels of APPLE0F000026657 were related to lower levels of fire blight for all three *E. amylovora *strains, while lower transcript levels of APPLE0FR00066754 and APPLE0FR00063520 were associated with lower levels of disease caused by Ea273 and Ea4001a, respectively (Additional File 2, Table S6).

## Discussion

In this study, we found that rootstock genotype influenced 'Gala' scion fire blight susceptibility in grafted apple trees. This indicates that at least some level of resistance possessed by the rootstock can be conferred upon the scion variety that is grafted to it. These phenotypic differences in scion fire blight susceptibility were associated with reproducible patterns of gene expression in uninfected trees. Most of the transcripts identified in this study had higher levels of expression in the least susceptible trees. The expression levels of some of these genes may play a role in determining the susceptibility status of apple trees to *E. amylovora *prior to infection. Some of the identified genes may also play a role in fire blight disease resistance after infection has begun. It is also possible that some of the genes identified in the study affect the suitability of the host environment for the bacterium, rather than being involved in defense directly.

Tree breeding is a slow and costly process, particularly due to long juvenile periods. The screening of seedlings for the expression of a suite of genes correlated with a given trait could provide a valuable short-cut to reduce breeding time. The suite of genes identified in the present study might be useful as predictors of the fire blight resistance status of apple trees and seedlings. Seedlings could be selected based on gene expression patterns associated with favorable traits. This approach is not novel; the use of expression-based markers has proven to be effective in the screening of human breast cancers in order to predict the aggressiveness of the tumor [28]. Some of the genes identified in this study might also be suitable targets for direct manipulation for improvement of apple tree fire blight resistance and for the development of sequence-based molecular breeding markers.

### Pathways and processes

Three transcripts on the array corresponding to genes in the phenylpropanoid pathway were on our preliminary list of rootstock-regulated candidates, including two for chalcone synthase (APPLE0F000017774, APPLE0F0000178640) and a chalcone isomerase (APPLE0F000056938) (Additional File 1, Table S2). However, these genes do not appear on any of our subsequent lists. Nevertheless, the expression pattern of the genes in the phenylpropanoid pathway as a whole (Figure 2) is consistent with the proposed role of this pathway in the response to *E. amylovora *infection [14,15]. This suggests that the expression of the phenylpropanoid pathway as a whole might be a good predictor of fire blight resistance.

Sorbitol dehydrogenase (SDH) (APPLE0F000058028, APPLE0F000007408, APPLE0F000017030), was found to be expressed at higher levels in the trees that were least susceptible to fire blight (Table 3 and Additional File 1, Table S4). Sorbitol is a major form of translocated sugar in apples [29]. SDH converts sorbitol to fructose in sink tissues [30]. For *E. amylovora*, sorbitol is an important factor in determining host specificity [31]. It may be that higher SDH levels reduce the availability of sorbitol to *E. amylovora*. However it has also been shown that high sorbitol levels can inhibit the development of disease symptoms [32]. Our analysis of the recently released apple genome suggests that there may be up to 28 genes encoding SDH enzymes, as opposed to a single copy of SDH in Arabidopsis (AT5G51970). This complexity points to the importance of SDH to apple physiology.

Not surprisingly, there was a GO annotation enrichment among the genes having higher expression in the least susceptible trees (Table 1), with genes predicted to be involved in responses to stress and biotic and abiotic stimuli being disproportionately represented relative to the known apple transcriptome as a whole. Upon further analysis of these transcripts (Table 2), more transcripts than expected, relative to the proportion among all of the transcripts represented on the array, were identified in the secretory pathway, including several heat shock proteins (APPLE0F000018523, APPLE0F000020273, APPLE0F000060823), suggesting that protein processing in the endoplasmic reticulum may be more active in the trees least susceptible to fire blight. Heat shock proteins are important for protein processing in the endoplasmic reticulum and have also been shown to play critical roles in signal transduction in defense responses in tobacco [33]. Additionally, higher levels of the transcripts encoding a predicted calnexin (APPLE0F000027501) and a Sec61 homolog (APPLE0F000020583) were associated with reduced fire blight susceptibility by stepwise multiple regression analysis. Both of these proteins have functional annotations indicating involvement in protein processing in the endoplasmic reticulum. Consistent with these findings, it has been shown that the entire secretory pathway is up-regulated during systemic acquired resistance (SAR) [34] as well as during the N-mediated viral defense response in tobacco [35].

Similarly, from the same data set, a greater than expected number of genes involved in both the endocytosis and peroxisomal pathways were identified, suggesting that these processes may be more active in the least susceptible trees. Intriguingly, endocytosis processes are involved in plant recognition of pathogen-associated molecular patterns (PAMPs) [36]. For example, the bacterial flagellin protein FLG22 is recognized in plants by the FLS2 receptor kinase [37], which leads to endocytosis of the FLS2 receptor and its subsequent degradation by the proteosome [38]. Higher levels of a transcript encoding a predicted fatty acyl CoA ligase (APPLE0F000019968) were associated with reduced fire blight susceptibility by the stepwise multiple regression analysis. The protein encoded by this gene has a functional annotation indicating involvement in endocytosis and fatty acid metabolism. We also identified a transcript encoding a putative phosphatidylinositol-4-phosphate 5-kinase (PIP5K) (APPLE0F000017691) that had higher expression levels in the least susceptible trees. The product of PIP5K, phosphoinositol 4,5-bisphosphate (PI(4,5)P2), is a key regulator of clathrin-mediated endocytosis [39].

### Additional candidate genes of particular interest

A major objective of this study was to identify candidate genes in apple that are potentially involved in determining fire blight resistance prior to an infection event. Interestingly, quite a few of the candidate genes identified in our study had previous links to disease resistance processes, including differential expression upon *E. amylovora *infection in apple [11-13]. This includes several heat shock proteins, a leucine-rich repeat transmembrane kinase, and sorbitol dehydrogenase. Strikingly, over half of the transcripts that we identified as being expressed at higher levels in less susceptible trees are down-regulated during *E. amylovora *infection [11-13]. It is possible that the expression of these genes is down-regulated by the pathogen to promote disease. Additionally, eight of the genes in both Tables 3 have also been shown to be phosphorylated upon infection [40], which offers another level of regulation in addition to changes in transcript abundance.

Only one gene was found in common between our data set and those of Norelli et al. [11], Baldo et al. [12], and Sarowar et al [13]. This gene had higher steady-state expression levels in resistant trees and was up-regulated in all three pathogen induction studies. The Arabidopsis homolog of APPLE0F000017734/APPLE0F000060312 (AT2G31880 or *SOBIR1*) (Tables 3 and 4, Additional File 1, Table S4) encodes a putative leucine rich repeat transmembrane protein that is expressed in response to *Pseudomonas syringae *infection in Arabidopsis. Overexpression of *SOBIR1 *in Arabidopsis caused a constitutive upregulation of *PR-1 *and *PR-2*, and the plants showed enhanced resistance to *P. syringae *DC3000, suggesting that elevated levels of *SOBIR1 *lead to a constitutive activation of disease-resistance responses [41]. *SOBIR1 *overexpression also resulted in the activation of cell death. It has been proposed that *SOBIR1 *may play a role in the regulation of the golgi apparatus, particularly during periods of cellular stress [42].

We also identified a transcript encoding a putative cell death regulator, inositolphosphorylceramide synthase (APPLE0F000021750, Additional File 1, Table S4), that had higher expression levels in the least susceptible trees. Inositol can be modified by inositolphosphorylceramide synthase to produce inositolphosphorylceramide. Inositolphosphorylceramide has been shown to be involved in the regulation of programmed cell death during the plant defense response [43].

Jasmonic acid has been shown to play an important role in the response to pathogens, in a pathway parallel to that of salicylic acid [44,45]. In particular, ethylene and jasmonate have been shown to play an important role in defense against necrotrophic pathogens like *E. amylovora *[46]. We also identified homologs of the *JAZ1 *gene in Arabidopsis (APPLE0F000019494/APPLE0F000070531, Table 3, Additional File 1, Table S4) that had greater expression in the least sensitive trees. The JAZ1 protein of Arabidopsis is part of the COI1/JAZ jasmonate receptor complex [47].

## Conclusions

The influence of rootstocks on the fire blight susceptibility and gene expression of the scion has allowed us to identify genes potentially associated with this phenotypic trait. The identification of these genes will contribute to the understanding of host-pathogen interactions as well as provide plant breeders with valuable new markers for improved disease resistance breeding. This study illustrates the utility of our rootstock-regulated gene expression data sets for candidate trait-associated gene data mining.

## Competing interests

The authors declare that they have no competing interests.

## Authors' contributions

TWM conceived the study and participated in its design and coordination. PJJ performed plant material preparation and mRNA extraction for microarray hybridizations. PJJ and NA carried out the computational analysis of the microarray data. NH and JWT conducted the fire blight susceptibility field study in Biglerville, PA. GF provided the tree samples and fire blight data from Geneva, NY. IM developed the contig sequences used for the microarray. CP and SNM assisted in the experimental design. CP performed the second-generation microarray hybridizations. RMC contributed to the experimental design, selection of rootstocks and general culture of trees in the field. HKN performed the multiple regression analysis of the gene expression data. PJJ and TWM analyzed the computational results and drafted the manuscript. All authors commented on the manuscript and approved the final version.

## Supplementary Material

Additional file 1 **Tables S1-S4**. **Table S1**. Expression patterns of putative phenylpropanoid genes from *Malus x domestica*. **Table S2**. Candidate fire blight resistance transcripts with higher expression in 'Gala' scion/rootstock combinations with lower susceptibility to E. amylovora. **Table S3**. Candidate fire blight resistance transcripts with higher expression in 'Gala' scion/rootstock combinations with higher susceptibility to E. amylovora. **Table S4**. Transcripts in common with those known to have differential expression in apple flowers upon E. amylovora infection [13] (Sarowar et al. 2011). Click here for file

Additional file 2 **Tables S5-S6. Table S5**. Stepwise multiple regression analysis parameter estimates and associated statistics for transcripts with higher expression in less susceptible trees. **Table S6**. Stepwise multiple regression analysis parameter estimates and associated statistics for transcripts with higher expression in more susceptible trees. Click here for file
