# Supplementary material for: Rootstock-regulated gene expression patterns associated with fire blight resistance in apple
Source: BMC Genomics. 2012 Jan 9;13:9. doi: 10.1186/1471-2164-13-9 (PMC3277459; doi:10.1186/1471-2164-13-9)
Supplement: Additional file 2 — Tables S5-S6. Table S5. Stepwise multiple regression analysis parameter estimates and associated statistics for transcripts with higher expression in less susceptible trees. Table S6. Stepwise multiple regression analysis parameter estimates and associated statistics for transcripts with higher expression in more susceptible trees. [file 1471-2164-13-9-S2.DOC]

| Table S5: Stepwise multiple regression analysis parameter estimates and associated statistics for transcripts with higher expression in less susceptible trees^a^. | | | | | | | | | | | | | | |
| --- | --- | --- | --- | --- | --- | --- | --- | --- | --- | --- | --- | --- | --- | --- |
|  | Ea 273^x^ | | | |  | Ea 2002^x^ | | | |  | Ea 4001a^x^ | | | |
| Gene | Parameter estimate^y^ | | *F*-Value | Pr > *F* |  | Parameter estimate^y^ | | *F*-Value | Pr > *F* |  | Parameter estimate^y^ | | *F*-Value | Pr > *F* |
| APPLE0F000019334 | 38.99 | | 6.87 | 0.013 |  | ---- | | ---- | ---- |  | 43.53 | | 3.15 | 0.084 |
| APPLE0F000018558 | 60.58 | | 32.47 | <0.001 |  | 41.59 | | 4.73 | 0.036 |  | ---- | | ---- | ---- |
| APPLE0F000060273 | -19.67 | | 23.30 | <0.001 |  | -25.77 | | 8.81 | 0.005 |  | -29.91 | | 13.99 | 0.001 |
| APPLE0F000020900 | -23.27 | | 3.67 | 0.064 |  | ---- | | ---- | ---- |  | ---- | | ---- | ---- |
| APPLE00R00018643 | -31.53 | | 9.14 | 0.005 |  | -34.6 | | 2.42 | 0.128 |  | ---- | | ---- | ---- |
| APPLE0F000020583 | 18.45 | | 5.44 | 0.026 |  | ---- | | ---- | ---- |  | 48 | | 13.22 | 0.001 |
| APPLE0F000019968 | -73.74 | | 30.31 | <0.001 |  | -99.55 | | 11.49 | 0.002 |  | ---- | | ---- | ---- |
| APPLE0F000023953 | 32.28 | | 11.38 | 0.002 |  | 51.09 | | 6.81 | 0.013 |  | ---- | | ---- | ---- |
| APPLE0F000016773 | -15.39 | | 2.43 | 0.128 |  | ---- | | ---- | ---- |  | ---- | | ---- | ---- |
| APPLE0F000017955 | ---- | | ---- | ---- |  | 45.42 | | 3.23 | 0.080 |  | ---- | | ---- | ---- |
| APPLE0F000027501 | ---- | | ---- | ---- |  | -25.07 | | 3.14 | 0.084 |  | -20.47 | | 2.95 | 0.094 |
| APPLE0F000015821 | ---- | | ---- | ---- |  | ---- | | ---- | ---- |  | 39.35 | | 6.5 | 0.015 |
| APPLE0F000021433 | ---- | | ---- | ---- |  | ---- | | ---- | ---- |  | 41.26 | | 7.55 | 0.009 |
| *R*^2^ (*P* -value)^z^ | | 68.96(<0.0001) | | | |  | 39.75(0.004) | | | |  | 48.66(0.0001) | | |
| ^a^Transcript expression levels were identified as being significantly related to the level of fire blight caused by three strains of *E. amylovora* on 48 apple trees based on stepwise multiple regression analysis. | | | | | | | | | | | | | | |
| ^x^Dashed lines indicate the genes were not identified as being related to fire blight levels caused by the strain. | | | | | | | | | | | | | | |
| ^y^Negative sign indicates upregulation of the gene is related to reduced levels of fire blight. | | | | | | | | | | | | | | |
| ^z^*R*^2^ = Percent of variance (*P*-value for an *F*-test for model significance) in fire blight severity accounted for by the model relating the levels of gene expression and an intercept to levels of fire blight. | | | | | | | | | | | | | | |

| Table S6: Stepwise multiple regression analysis parameter estimates and associated statistics for transcripts with higher expression in more susceptible trees^a^. | | | | | | | | | | | | |
| --- | --- | --- | --- | --- | --- | --- | --- | --- | --- | --- | --- | --- |
|  |  | Ea 273^x^ | | |  | Ea 2002^x^ | | |  | Ea 4001a^x^ | | |
| Gene | Function | Parameter estimate^y^ | *F*-Value | Pr > *F* |  | Parameter estimate^y^ | *F*-Value | Pr > *F* |  | Parameter estimate^y^ | *F*-Value | Pr > *F* |
| APPLE0F000026657 |  | 48.89 | 5.61 | 0.023 |  | 60.82 | 3.82 | 0.0571 |  | 52.41 | 3.34 | 0.074 |
| APPLE0FR00081295 |  | -19.58 | 2.63 | 0.113 |  | ---- | ---- | ---- |  | ---- | ---- | ---- |
| APPLE0FR00067567 |  | -47.39 | 7.03 | 0.012 |  | ---- | ---- | ---- |  | -69.41 | 6.28 | 0.016 |
| APPLE0FR00066754 |  | 33.61 | 4.64 | 0.038 |  | ---- | ---- | ---- |  | ---- | ---- | ---- |
| APPLE0F000016771 |  | -26.01 | 3.06 | 0.088 |  | ---- | ---- | ---- |  | ---- | ---- | ---- |
| APPLE0FR00047902 |  | ---- | ---- | ---- |  | -40.92 | 2.55 | 0.118 |  | ---- | ---- | ---- |
| APPLE0F000061746 |  | ---- | ---- | ---- |  | -5619 | 6.41 | 0.015 |  | ---- | ---- | ---- |
| APPLE0FR00063520 |  | ---- | ---- | ---- |  |  |  |  |  | 40.89 | 4.58 | 0.038 |
| *R*^2^ (*P* -value)^z^ | | 32.06(0.009) | | |  | 29.79 (0.020) | | |  | 28.89 (0.031) | | |
| ^a^Transcript expression levels were identified as being significantly related to the level of fire blight caused by three strains of *E. amylovora* on 48 apple trees based on stepwise multiple regression analysis. | | | | | | | | | | | | |
| ^x^Dashed lines indicate the genes were not identified as being related to fire blight levels caused by the strain. | | | | | | | | | | | | |
| ^y^Negative sign indicates upregulation of the gene is related to reduced levels of fire blight. | | | | | | | | | | | | |
| ^z^*R*^2^ = Percent of variance (*P*-value for an *F*-test for model significance) in fire blight severity accounted for by the model relating the levels of gene expression and an intercept to levels of fire blight. | | | | | | | | | | | | |
